# Lessons on the COVID-19 pandemic, for and by primary care professionals worldwide

**DOI:** 10.1080/13814788.2020.1820479

**Published:** 2020-09-28

**Authors:** Salman Rawaf, Luke N. Allen, Florian L. Stigler, Dionne Kringos, Harumi Quezada Yamamoto, Chris van Weel, On behalf of the Global Forum on Universal Health Coverage and Primary Health Care

**Affiliations:** a WHO Collaborating Centre, Department of Primary Care and Public Health, Imperial College London, London, UK; b Nuffield Department of Primary Care Health Sciences, University of Oxford, Oxford, UK; c Institute of General Practice and Evidence-based Health Service Research, Medical University of Graz, Graz, Austria; d Department of Public Health, Amsterdam UMC, University of Amsterdam; and Amsterdam Public Health Research Institute, Amsterdam, Netherlands; e Department of Primary and Community Health, Radboud University Medical Center, Nijmegen, The Netherlands; f Department of Health Services Research and Policy, Australian National University, Canberra, Australia

**Keywords:** COVID-19, pandemic, primary health care, health system, virtual focus group

## Abstract

The COVID-19 pandemic has modified organisation and processes of primary care. In this paper, we aim to summarise experiences of international primary care systems. We explored personal accounts and findings in reporting on the early experiences from primary care during the pandemic, through the online *Global Forum on Universal Health Coverage and Primary Health Care.*

During the early stage of the pandemic, primary care continued as the first point of contact to the health system but was poorly informed by policy makers on how to fulfil its role and ill equipped to provide care while protecting staff and patients against further spread of the infection. In many countries, the creativity and initiatives of local health professionals led to the introduction or extension of the use of telephone, e-mail and virtual consulting, and introduced triaging to separate ‘suspected’ COVID-19 from non-COVID-19 care. There were substantial concerns of collateral damage to the health of the population due to abandoned or postponed routine care.

The pandemic presents important lessons to strengthen health systems through better connection between public health, primary care, and secondary care to cope better with future waves of this and other pandemics.

## Introduction

KEY MESSAGES

- Covid-19 has had a complex impact on primary care, with improved access and coordination in many settings, balanced against resourcing and information flow issues, and a reduction in the comprehensiveness of services.
- Primary care remains the cornerstone of pandemic response and has shown itself to be highly adaptable in meeting the unique demands of the pandemic.
- Primary care needs to be resourced, with sufficient equipment, training, and financing.

The COVID-19 pandemic started in Wuhan, China in December 2019 and spread to more than 200 countries and territories, by the end of April 2020 causing more than two million confirmed cases and more than 150,000 confirmed deaths [1]. The pandemic, officially characterised on 11^th^ March 2020, had an immediate and profound effect on societies and brought social and economic life to a standstill. At the same time, it required adaptations of countries’ health systems and urged primary care and hospitals to respond to challenges in new health needs and requirements by changing organisation and processes more rapidly than ever before.

Although a lot of attention has been focussed on hospital care, and in particular the role of intensive care units, this was only the tip of the iceberg. Only a small percentage of coronavirus patients are being hospitalised around the world. Primary care, as the first contact with the health system, is managing the main share of COVID-19 related care and will be the ‘first in and last out’ [2,3]. This includes managing the aftermath of the pandemic and the collateral damage caused by reallocating resources to COVID-19 whilst inadvertently hindering access to a wide range of primary care service.

The response to the pandemic has changed the ways primary care will operate. In this paper we aim to summarise the experiences of international primary care systems and highlight lessons learned related to positioning and supporting primary care in the context of the overall health systems to cope with future pandemic challenges.

## The search for experiences

Taking into account the urgency and lack of immediate fund, the authors have explored personal accounts and findings on the early experiences from primary care during the COVID-19 pandemic virtually among members of the *Global Forum on Universal Health Coverage and Primary Health Care.* This forum connects Universal Health Coverage (UHC) and Primary Healthcare (PHC) experts from all over the world. The aims are to learn and to support each other by sharing experiences and information. Participants are clinicians, academics and policy makers that currently sum 410 members from 68 countries. Through snowballing, some members were contacted and were asked the following questions: How COVID-19 has influenced the functioning of health systems and the role of primary care within it?; What are the ways that primary care has changed and adapted (re-designed) its organisation to respond to the needs of local populations?; Are there any changes in health problems that practices have encountered?; Can you record changes in behaviour of patients with regards to their health problems?; Have you observed changes in health outcomes? Out of 410 members from 68 countries, 54 members from 29 countries responded (17 members from Europe, 15 from Asia-Pacific, 13 from the Americas, 8 from Africa and 1 member from Eastern Mediterranean) and shared the experiences of their colleagues and teams, as well as personal account, during the pandemic. Members of the group also shared many supporting documents. This qualitative opinion exercise, despite its limitation, provided an excellent platform for the Group to exchange their experience and document some of the innovative and creative approaches in general practice yet to be fully recognised, during the early weeks of the unprecedented COVID-19 pandemic.

## How COVID has influenced the functioning of health systems and changed the role of primary care

Colleagues from the Global Forum shared that during the pandemic, primary care continued to function in many countries as the first point of contact for a broad spectrum of patients: (i) those with suspected COVID-19; (ii) patients with test-confirmed COVID-19 (with or without symptoms); (iii) patients in contact with suspected or confirmed case(s) but without symptoms, (iv) patients anxious about COVID-19, and (v) patients with non-COVID-19 health needs. With the exception of Taiwan, Singapore, Hong Kong and South Korea who had learned valuable lessons from the SARS and MERS epidemics, virtually all other countries were caught ill prepared [4]. Furthermore, in the critical early phase there was a lack of coordination between different sectors of domestic health systems. Access to primary care was partly hindered due to the suspension or postponing of service provision, and partly due to a reduction in care-seeking behaviour – prompted both by patient anxiety around becoming infected and by coordinated messaging and educational campaigns encouraging patients to avoid using health services where possible. Although full attention at the community level was required to prevent spread of the virus, the hospital sector was prioritised in the supply of personal protective equipment (PPE) and testing facilities. The percentages of health and social care workers’ absence from work due to unsafe practices or fear of infecting family members were rising rapidly around the world and have been reported as high as 40%. Primary care has also stepped in to help other sectors by expanding its scope of practice and loaning staff to work in hospitals and other sectors. For instance, in Spain it has become common practice for primary care providers to look after patients in nursing homes [5].

## The way primary care changed and adapted (re-designed) its organisation to respond to the needs of the population

Primary care professionals in different parts of the world have initiated ‘on the spot’ innovative approaches to allow for ‘medical distancing’ in almost all countries, while continuing the delivery of care through virtual consultation and monitoring, and the use of patient apps where possible. In some countries (e.g. US, Canada) and for certain patient groups (e.g. those with mental health needs) the idea of conducting consultations remotely was inconceivable before the pandemic [6–9]. Unfortunately, in many resource-poor settings (Africa, Latin America) the options to move to virtual consulting have been much more limited. Laws and practice guidelines are often being rapidly adjusted and reimbursement codes are developed to overcome the barriers of telehealth in an effort to reduce healthcare-related COVID-19 transmissions, and to protect health personnel [10]. In the Netherlands, additional financial support of primary care was swiftly arranged, and there was a substantial improvement in the direct access in hospitals to primary care electronic patient records [11].

By our reckoning, ‘distanced’ contacts could currently account for more than half of all patient contacts in primary care, and research has shown this to be the case in the Netherlands [12]. This ability to initiate new approaches of care should be evaluated and where appropriate maintained as positive signs of the responsiveness of primary care.

Such a change in practice organisation enabled family physicians and teams to reduce their risk of exposure at work whilst prioritising care through triage. Using tele-medicine, patients with suspected COVID-19 symptoms did not have to attend the practice but were able to call, email or video-consult for advice, home assessment or – in case of severe symptoms – could be directed for hospital care. A variation of this was the direction of patients with high suspicion of COVID-19 (those with fever, cough and/or close COVID-19 contacts) to telemedicine services or designated ‘hot’ community clinics staffed by primary care professionals enabling regular primary care clinics to see lower-risk patients. Other practices have redesigned the flow of COVID-19 and non-COVID-19 cases by segregating practice hours and/or separating areas in the practice, accessible through different entrances.

A major problem has been the availability of PPE including medical masks. Under the prevailing global shortage PPE has been disproportionately allocated to hospitals at the expense of primary care and nursing homes. Some family physicians have withdrawn their service because of the lack of adequate PPE [13]. It is also worth noting that just as hospitals have tended to suck PPE away from primary care, high-income countries have quickly moved to supply their health systems, creating procurement issues for low- and middle-income countries with less well-developed supply chains.

## The change in health problems encountered by practices

Within a short period of time a sharp decline in non-COVID-19 contacts was observed in many countries, combined with an increase of acute respiratory and COVID-19 related health problems to the degree that the latter accounted for the majority of problems encountered [12]. This resulted in postponement of ‘regular’ care – with risk of collateral damage due to loss of contact with vulnerable groups (including frail elderly, ‘illegal’ migrants, those with low literacy, victims of domestic violence, homeless populations, pregnant women and young children) and lack of care provision, in particular for patients with multiple chronic health problems. Social distancing, and fear about becoming infected are likely to have contributed to this decline. In addition, the COVID-19 measures of physical distancing, quarantine, and lock down have an impact on social life, thereby generate new health problems, that increase the need of care.

## The behaviour of patients concerning their health problems

Most Governments and professional bodies were quick to disseminate messaging to relieve pressure on services by staying away from primary care unless one absolutely had to. To a large extent this messaging changed behaviours successfully and the general public has stayed away. Patient anxiety around contracting the illness at health centres may have also contributed to a noticeable drop in appointments in many countries. In most countries, people were told that they could only leave their homes for five reasons (healthcare, essential shopping, caring for others in need, work if one cannot work from home, and exercise). In some countries the criteria did not include leaving the house for urgent or important healthcare needs.

Our colleagues have the impression that patients are ‘storing up’ problems and we might be met with an increase in demand when restrictions are eased. It is not yet clear how substantial and urgent the demand for postponed care will be, but gradually policy makers and professionals are beginning to consider this issue and starting to advise the public to contact their family physician or hospital for non-COVID related health needs.

## Changes in health outcomes

The decline in non-COVID-19 related care raised concerns of the quality of essential care of patients with chronic health problems, for example, the pro-active care of diabetes. We do not yet have data on the impact of suspended routine care. A substantial drop in hospital treated myocardial infarctions has been documented in Austria [14]. It is likely that these deaths are still occurring, but not reaching secondary care. Indeed, recent data from the UK has shown that although registered all-cause mortality has been running lower than normal for the past few months, in mid-April there was an increase in excess mortality from all-causes [15].

Finally, population information on when and how to contact primary care and what patients can do themselves have proven their value [16]. Primary care has a key role to play during and after the pandemic by using its information infrastructure to identify risk groups, monitor compliance, provide care according to needs, and to detect new cases of COVID-19 infections. COVID-19 may have provided a unique policy window to convince governments to enforce better public health policies [17].

## The overall impact

Beyond primary care, COVID-19 brought relevant lessons for health policy and health systems at large. In the context of this analysis we identified *five* points. The *first* is the need to come to a full understanding of importance of primary care in responding to a pandemic and value its power to swiftly adapt to circumstances it had never before experienced while maintaining a strong rapport with local practice populations. The *second* is the importance to support professionals in primary care to cope with the stress and strains of working under conditions of a pandemic, through balanced work scheduling and collaborative working relationship in defined geographical areas. As a *third,* it is essential to protect primary care services and to make sure that such services are provided to all those who need it. The *fourth* point is that the COVID-19 experience stresses the importance of interaction between primary care and public health. The *fifth* point is that the experiences collected during the COVID-19 pandemic should find their way into the teaching and training of health professionals. As COVID-19 had an impact on health systems in all their components, this makes it particularly attractive to address professional needs through multidisciplinary teaching and training programmes – involving public health, primary and hospital care, from nurses, physicians to allied health professionals together. Responding to pandemics requires international collaborations, thus it would be appropriate that teaching and training programmes could also operate in an international context.

## Conclusion

The final aftermath of the different approaches of support versus disincentive of primary care, and whether strong primary care resulted in lower COVID-19 hospitalisations is yet to be confirmed. It is important that hard-won lessons are retained and lead to better preparedness in future pandemics. For primary care, in its role as the first contact with the health system, there are at least three major learning points:

*First*, primary care has been able to rapidly change and innovate with redirecting patient flow. Being able to do so has been a major concern. However, at the same time, it is where primary care, often with little or no external support, has achieved a lot of success. In this respect Singapore was exemplar, with community health centres that have fully equipped consulting rooms that can be isolated from the rest of the practice, and a separate entrance accessible by ambulance and stretcher.

The *second* lesson is the importance of maintaining access to primary care and ongoing management of all health problems that impact the local population. This means the planning and organisation of ‘regular’ primary care for a large majority of health needs in all individuals, in particular to those in highest need, parallel to addressing needs specific to the epidemic. The COVID-19 lesson here was that it was fairly easy to convince the population to postpone their contacts with primary care in the first days when practices had to carry out a reorganisation to the new situation. The bigger problem was encouraging people to resume seeking care as usual as quickly as possible.

The *third* lesson is related to the collection and dissemination of finely tuned information that can be fit for purpose. Users of information (public, patients, clinicians, or policymakers) all have their own information needs that cannot be served by the same measures. The general public is currently overwhelmed with epidemiological data disseminated by the media, often with little sense of their quality, caveats, risk adjustment issues etc. Effort should be put into addressing information needs of the public in a comprehensible manner, that takes notion of people’s fear of the spread of the virus and to counter ‘fake news’ or data that leaves room for misinterpretation, that in all probability will accompany each new pandemic.
